# Non-Averaged Single-Molecule 3D Structures Capture RNA Maturation Intermediates by Individual-Particle Cryo-Electron Tomography

**DOI:** 10.1063/4.0000897

**Published:** 2025-10-27

**Authors:** Gang Ren, Jianfang Liu, Meng Zhang

**Affiliations:** Lawrence Berkeley National Laboratory, Berkeley, CA, USA

## Abstract

Understanding the structural dynamics of biomolecules at the single-particle level is essential for elucidating molecular functions, folding mechanisms, and conformational transitions that underlie biological processes. RNA molecules, in particular, follow complex folding pathways to achieve their functional tertiary and quaternary structures. These pathways are characterized by rugged energy landscapes populated by multiple transient intermediates, many of which are too short-lived or heterogeneous to be resolved by traditional structural methods. Conventional approaches such as X-ray crystallography, nuclear magnetic resonance (NMR) spectroscopy, and single-particle cryo-electron microscopy (cryo- EM) provide high- resolution structures but generally yield static, ensemble-averaged models derived from thousands or millions of particles. This averaging process masks conformational heterogeneity and dynamic intermediates that are critical for understanding RNA folding and maturation.

To address this challenge, our team has advanced cryo-electron tomography (cryo-ET) (**Fig. 1a**), traditionally used for low-resolution imaging of large cellular structures, adapting it for single-molecule structure determination at intermediate resolution (∼2–10 nm). This method, termed individual-particle electron tomography (IPET), allows direct three-dimensional reconstruction of single biomolecules without particle selection, classification, or averaging. IPET preserves molecular heterogeneity, enabling visualization of conformational states and intermediates in their native environment.

IPET incorporates several key technological innovations: Constrained iterative reconstruction algorithms (Zhang et al., *PLoS One*, 2012, 7(1):e30249), which improve reconstruction accuracy from limited tilt data; Image contrast enhancement techniques (Wu et al., *Sci. Rep.*, 2018, 8(1):16711), which increase signal-to- noise ratios under low-dose imaging conditions; Automated tilt-series acquisition systems (Liu et al., *Sci. Rep.*, 2016, 6:29231), improving throughput and reproducibility; Missing-wedge correction algorithms (Zhai et al., *Sci. Rep.*, 2020, 10:10489), recovering critical angular information and mitigating anisotropic distortions. Together, these innovations allow us to push cryo-ET resolution toward ∼2 nm for single particles, despite challenges associated with low signal intensity and radiation damage. In this study, we applied IPET to capture the self-folding process of RNA origami nanoparticles, revealing intermediate 3D structures along their maturation pathway with tertiary structure-level resolution (Liu et al., *Nat. Comm.*, 2024, 15:9084). The RNA nanoparticles were engineered to fold into a well-characterized six- helix bundle with a clasp helix (6HBC). We optimized cryo-ET data acquisition parameters to maximize structural integrity while minimizing electron beam-induced damage, avoiding chemical fixation, particle selection, or averaging to ensure that each reconstruction represented the native conformation of an individual molecule (**Fig. 1b–f**).

We reconstructed 120 individual RNA particles, resulting in 120 distinct 3D density maps (**Fig. 1g**). These reconstructions displayed remarkable heterogeneity, with each particle showing unique tertiary arrangements and helix configurations. Statistical analysis, including hierarchical clustering based on root-mean-square deviation (RMSD), confirmed two previously reported conformational states ("young" and "mature" structures, PDB: 7PTK and 7PTL). Importantly, we also identified multiple novel intermediates and hyper-compact configurations that had not been previously observed (**Fig. 2a, b**). These findings support a multi-step maturation pathway characterized by progressive helix-helix compaction, structural rearrangements, and localized flexibility. The ability to directly observe these intermediates provides critical experimental validation for long-standing theoretical models of RNA folding landscapes (**Fig. 2c**).

Beyond RNA, IPET has proven broadly applicable to studying other flexible macromolecular complexes. For example, we have applied IPET to characterize the conformational transitions of tetra-nucleosome arrays during phase separation, which are fundamental to chromatin compaction and transcription regulation (Zhang et al., *Nat. Comm.*, 2024, 15:4395). These studies revealed that chromatin condensation is driven by shifts in the DNA entry-exit angle relative to the nucleosomal disc, and that phase transitions expose hydrophobic nucleosome surfaces, thereby modulating inter-nucleosome interactions (Zhang et al., *Mol. Cell*, 2022, 82(16):3000–3014.e9). This insight advances our understanding of chromatin organization and its dynamic transitions between interphase and metaphase.

The implications of IPET extend beyond experimental structural determination into computational modeling and artificial intelligence (AI). Large datasets of single-particle 3D structures capturing a variety of conformational states provide high-quality training sets for AI models designed to predict folding pathways, structural dynamics, and biomolecular functions. Statistical analysis of hundreds of individual reconstructions enables the construction of experimentally derived conformational landscapes (**Fig. 2c**), offering new data to refine molecular dynamics simulations and improve predictive models in AI-driven structural biology. Furthermore, the ability to visualize transient, low-population states has significant implications for drug discovery, revealing cryptic binding sites and allosteric pockets not detectable in averaged structures.

In conclusion, IPET represents a transformative step forward in single-molecule structural biology, enabling the direct visualization of 3D structural snapshots of individual macromolecules in their native, dynamic states. In this work, we demonstrated the power of IPET to elucidate the complex conformational landscape of RNA during its maturation process, uncovering previously inaccessible intermediates and highly compact states. These findings not only advance our understanding of RNA folding mechanisms but also highlight IPET potential to contribute to the study of other dynamic systems, including antibody, chromatin, lipoprotein, Coronaviruses, and large protein assemblies. As the IPET platform continues to evolve and integrates with AI and computational simulation tools, it is poised to become an indispensable tool for understanding biomolecular dynamics at an unprecedented level of detail, with broad applications in structural biology, synthetic biology, pharmaceutical development, and precision medicine.

